# A Review of the Latest Advances in Encrypted Bioactive Peptides from Protein-Rich Waste

**DOI:** 10.3390/ijms17060950

**Published:** 2016-06-16

**Authors:** Ailton Cesar Lemes, Luisa Sala, Joana da Costa Ores, Anna Rafaela Cavalcante Braga, Mariana Buranelo Egea, Kátia Flávia Fernandes

**Affiliations:** 1 Federal University of Rio Grande, Chemistry and Food School, Rio Grande 96201-900, Brazil; ailtonelemes@hotmail.com (A.C.L.); luisasalaea@gmail.com (L.S.); joanaores@gmail.com (J.d.C.O.); 2 Federal University of São Paulo, Department of Bioscience, Santos 11015-020, Brazil; annarafaela@gmail.com; 3 Federal Institute of Education, Science and Technology Goiano, Campus Rio Verde, Rio Verde 75901-970, Brazil; 4 Federal University of Goiás, Institute of Biological Sciences II, Goiânia 74001-970, Brazil; kfernandes.lqp@gmail.com

**Keywords:** peptides, bioactivity, residual waste, purification, nanotechnology

## Abstract

Bioactive peptides are considered the new generation of biologically active regulators that not only prevent the mechanism of oxidation and microbial degradation in foods but also enhanced the treatment of various diseases and disorders, thus increasing quality of life. This review article emphasizes recent advances in bioactive peptide technology, such as: (i) new strategies for transforming bioactive peptides from residual waste into added-value products; (ii) nanotechnology for the encapsulation, protection and release of controlled peptides; and (iii) use of techniques of large-scale recovery and purification of peptides aiming at future applications to pharmaceutical and food industries.

## 1. Introduction

Bioactive peptides are compounds that exhibit an effect on body functions or conditions and may influence human health [1]. They differ widely in their amino acid composition, chemical structure and, therefore, in their biological function. These compounds frequently have cholesterol-lowering effects, besides antiprotozoal, antiviral, antithrombotic, antioxidant, antihypertensive and antimicrobial activities [2,3,4,5], which make them attractive for application to foods and pharmaceuticals.

Bioactive peptides may contain from three to 20 amino acid residues per molecule. They may be free or encrypted within the protein sequence. Encrypted peptides become active when released from the protein sequence [6,7] mostly by acid and alkaline chemical hydrolysis [8], proteolytic action of microorganisms or enzymatic hydrolysis of proteins. However, the last two are most widely used for food and pharmaceutical industrial applications [9].

Most reported peptides are derived from expensive protein matrixes (e.g., food), which in most cases make their application unfeasible. Processes that do not result in negative environmental impacts (green processes) have currently stood out since they aim at the use of agricultural waste to replace non-renewable resources. Waste generated by agro-industries is a protein-rich source and has become an alternative for obtaining compounds with bioactivity, mainly from protein hydrolysates.

After bioactive peptides have been obtained and have proven their power to act, they can be applied to food or drugs as bioactive agents. However, for commercial use, these compounds must be purified. To explore their physicochemical properties and evaluate their bioactivities the downstream process of peptides with functional properties are also very important [10].

In addition, the activity of bioactive peptides can be reduced by their susceptibility to proteolytic degradation or undesirable interactions with other compounds. Since most of the biological processes occur at the nanoscale, nanoparticulate technology has a promising future in developing novel preventive, diagnostic and therapeutic agents such as bioactive peptides.

The use of nanoparticles for peptides encapsulation is very important to protect these biomolecules [11], thus, providing higher therapeutic efficacy with progressive and controlled release of the drug during the degradation of the matrix [12]. Encapsulation may also allow the development of delivery systems [13] by improving stability [11], increasing residence time in the circulation and significantly decreasing toxicity [12].

This review concerns the novel approach for producing bioactive peptides from protein-rich waste. Some issues, such as recovery, purification process and nanoencapsulation for efficient industrial-scale production of bioactive peptides, have also been highlighted.

## 2. Peptides

The role of proteins has been widely known as physiologically active components in the diet. In addition, proteins have regions inside the molecule that perform both the protection and regulation of biological functions. These specific protein fragments, which are denominated peptides, may have a positive effect on body functions or conditions and affect human health positively [1,14].

Peptides can naturally occur in raw food materials and exert their physiological action directly. The synthesis of natural peptides may occur through ribosomal and nonribosomal mechanisms [15]. Furthermore, peptides can occur in an encrypted form, in which the bioactive molecule is inactive or in latent form within the sequence of the protein, and can be released and activated by proteolysis or several other techniques [16]. Bioactive peptides affect numerous biological processes, thus yielding behavioral, neurological, hormonal, nutritional, gastrointestinal effects, hyperglycemic and anti-tumor activities [17,18,19,20,21,22].

Despite their positive effect on body functions, a few peptides, such as the ones obtained from Amanita (fungal basidiomycetes), were identified as producers of toxic effects on a variety of cells; their ingestion can lead to death [23]. Furthermore, other toxic peptides were associated to the formation of toxic aggregates in Alzheimer's disease [24,25] and celiac disease [26,27], among others.

In this review, just encrypted peptides with positive effect on body functions will be discussed because of their potential low cost and ease to be obtained from low cost raw material which could cause environmental impact if it were disposed of inappropriately.

Peptides may contain from three to 20 amino acid residues per molecule [6,7] and differ widely in their chemical structure; hence, in their biological function. However, they have some characteristics in common: they are organic substances, usually of low molecular mass and have protective action on health when they are found in the diet in significant amounts.

In addition, they may have antiprotozoal, antiviral and antithrombotic activity, thus, reducing cholesterol levels and body mass [9,28], and even antioxidant, antihypertensive and antimicrobial activity [2,3,4,29,30,31,32], which makes them attractive for application to foods and pharmaceuticals.

The commercial market for peptides and proteins drugs has been estimated to be >$40 billion/year [33]. Annual sales of peptide drugs are growing at accelerated pace, about $20 billion/year, corresponding approximately 2% of the huge drug market [34].

Encrypted peptides can be found in animal and plant sources, such as milk, dairy products, eggs, fish, oysters, cereals (rice, wheat, buckwheat, barley and corn), soybeans, radish seeds [3,35,36,37] and other protein-rich sources.

Antioxidant peptides may be applied to oxidative processes since the oxidation results in the production of free radicals (O$\begin{array}{c} -\\ 2 \end{array}$, OH, H_2_O_2_) during the metabolism and respiration in aerobic organisms. When they are produced in excess and are not eliminated, free radicals can attack the nearest molecules by subtracting electrons and starting a chain reaction in which a molecule without an electron attacks other molecules, and so on [38]. Free radicals play a critical role in health-related disorders and can lead to heart disease, atherosclerosis, diabetes, cancer and neurological diseases [39]. In food, they may result in the deterioration of quality attributes such as flavor, color and texture [40].

The antioxidant activity of peptides is related to their composition, hydrophobicity, structure and ability to delay or prevent oxidative processes from the donation of electrons, with stabilization of the free radical, which remains in the structure of antioxidant rather than in the reaction. Antioxidant peptides have been produced by hydrolyzing various protein substrates such as fish proteins, soybeans, marine algae and a variety of dairy products [41,42,43,44,45].

Antimicrobial peptides act against a wide variety of pathogenic microorganisms, such as bacteria, fungi and viruses [46]. The action mechanism of antimicrobial peptides generally involves changes in biological membranes; it initially occurs by electrostatic attraction between molecules of peptides, usually positively charged, and anionic lipids found on the membrane surface. Then, due to the amphipathic structure of these peptides, interaction between the peptides and the membrane surface occurs [47,48], with degradation of the microbial cells by formation of ion channels or by production of transmembrane pores. This process causes an imbalance of cellular contents, thereby regulating the process of replication, transcription and translation of the DNA sequence by binding to specific intracellular targets, preventing the multiplication and growth of microbial cells [49].

The role of antihypertensive peptides in the regulation of blood pressure is related to the inhibition of the angiotensin I-converting enzyme (ACE) [50]. Inhibitors of this enzyme are, in fact, one of the main alternatives for the treatment of hypertension [51]. An alternative for the use of synthetic inhibitors against ACE would be their replacement by natural encrypted peptides capable of promoting the inhibition of the enzyme. Most of the peptides with ACE inhibition activity have short sequences in the range from two to 12 amino acids. The most effective antihypertensive peptides contain hydrophobic amino acids such as proline, especially in the C-terminal position, or positively charged amino acids, such as lysine and arginine at a terminal position [52].

Antihypertensive peptides have been obtained from several sources: dairy products [53], microbial cultivations with the bacterium *Oenococcus oeni* in wine protein substrate [54], enzymatic hydrolysis of proteins from *Parkia speciosa* seeds [55], from the use of hydrolyzed bovine lactoferrin derived from whey protein milk [56], enzymatic hydrolysis of protein concentrates recovered by ultrafiltration from cuttlefish processing wastewaters [57] and water soluble peptide extracts from dry-cured ham [58].

Opioid peptides can be considered compounds which may exert effects on the nervous system, similar to opium (morphine). Hydrolysates generated by the action of digestive enzymes in casein substrate have generated peptides with opioid properties [59,60]. The mechanism of peptides with opioid property appears to be related to their property receptor ligand, which has agonistic or antagonistic activities. Opioid receptors located in different parts of mammals can interact with endogenous ligands, exogenous opioids and opioid antagonists. The opioid receptors involved in this mechanism are present in the gastrointestinal tract, endocrine, nervous and immune systems [61,62].

Thus, when opioid peptides are orally administered, they can modulate absorption processes in the gut and influence the functioning of the gastrointestinal system in two ways: (1) affecting smooth muscles, a fact that results in limited intestinal transit time; and (2) affecting electrolytes transport of electrolytes. The effects of these compound derived from milk still need confirmation for adult consumers [63], since in newborn the opioid function of encrypted peptides from casein fractions is well known. Several peptides with different properties can be obtained and applied to foods and drugs after having their bioactivity evidenced. Therefore, constant study is required to obtain technology so that real application can be carried out.

## 3. Obtaining Peptides from Agro-Industrial Waste

Continued development of bio-sustainable and renewable resource technologies is extremely important with respect to environmental concerns [64], since the significant progress in agribusiness resulted in increased consumption of inputs and, consequently, in an increase in the generation of waste in agro-industrial activities.

The use of agro-industrial waste involves the use of materials generated as by-products, since the total volume thereof is large [65] and when they are not used, they raise the costs of disposal to prevent environmental pollution [66]. In this sense, there is growing interest in developing processes that enable the total use of waste [67]. Processes that do not result in negative environmental impact (green processes) have currently stood out since they aim at the use of agricultural waste to replace non-renewable resources.

Waste generated by the agro-industries is a protein-rich source and has become an alternative for obtaining compounds with bioactivity, mainly from protein hydrolysates. After bioactive peptides have been obtained and have proven their power to act, they can be applied to food or drugs as bioactive agents. Apart from creating potential environmental problems, waste represents loss of raw materials and energy, thus requiring significant investments in treatments for pollution control [68].

Waste can contain many valuable substances and through a suitable process or technology, this material can be converted into value-added products or raw materials that can be used in secondary processes [69].

**Figure 1:**
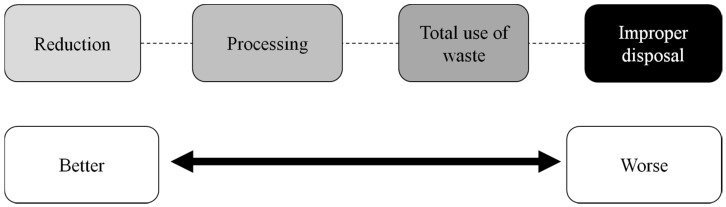
An ideal process should avoid generation of waste. Adapted from Pelizer, Pontieri and Moraes [68].

The current trend is to employ production processes that do not harm the environment or that can reduce the release of waste, considering that the best process is the one that reduces generation of waste and the worst process is the one that generates waste, which is improper for disposal (Figure 1).

Several bioactive peptides produced by enzymatic hydrolysis of various food proteins have recently been shown to possess bioactivity [70,71,72,73]. By-products and waste represent a relatively cheap source, thus, their use for the production of bioactive peptides will not only result in the reduction of production costs, highly significant to the development of added-value nutritional by-products but also in the mitigation of the problem regarding waste disposal [74].

Large amounts of protein-rich waste and bioactive nitrogenous compounds have extensively been generated every year. The major waste and by-products generated in the agro-industrial activity comprise microalgae [75,76], soybean meal [77], residues of olive production [78], rapeseed meal [79], chicken feathers [80], fish waste [81] and egg protein [82]. In particular, this waste is proteinaceous in nature and includes proteins, peptides and amino acids. Furthermore, protein-rich sources are ideal materials for bioactive peptide generation [8]. Some waste generated in large amounts and used for obtaining bioactive peptides is shown in Table 1.

Alga protein waste is a by-product derived from the water-extraction process of microalgae manufacturing, e.g., during alga essence production. The underused alga waste, with over 50% protein, has low economic value since it is normally used as animal feed [75]. As an example, more than 100 tons of alga protein waste has been harvested every year in Taiwan [87]. Thus, the bioconversion of alga protein waste into bioactive compounds can add value to this waste.

Peptides from *Chlorella vulgaris* alga waste, which were hydrolyzed by pepsin, showed high efficiency in scavenging various free radicals [75]. In addition, the authors reported that peptides from alga protein waste presented antioxidative *in vitro* potential. Thereby, these bioactive peptides can be used for the prevention of oxidative stress-related diseases.

Cian, Alaiz, Vioque and Drago [76] obtained bioactive peptides from algal residual cake (*Porphyra columbina*) with ACE inhibitory activity and antioxidant properties. To obtain theses peptides, a cellular rupture step is necessary; that is, when the cell wall is disrupted, the intracellular components are released, including proteins.

Potent antioxidative peptide was obtained from the hydrolysis of alga protein waste. Alga protein waste was digested by pepsin at an enzyme to substrate ratio of 2% (*w*/*w*) at 50 °C for 15 h. The peptide was isolated by sequential ammonium sulfate precipitation, gel filtration and ion exchange chromatography. The isolated peptide can degrade different free radicals, such as hydroxyl, superoxide, peroxyl, DPPH and ABTS radicals. In addition, the isolated peptide has anticancer activity [75].

Similarly, during fish processing, the fishing industry generates large amounts of by-products, which are destined especially for the manufacture of flour or even discarded into the environment [88]. Thus, bioactive peptides from marine processing waste and shellfish are also very promising as functional food ingredients. The waste derivate from fish and shellfish represent a potential source of biofunctional peptides, since it contains high quality and high levels of proteins, around 10%–23% (*w*/*w*) [8].

Robert, Zatylny-Gaudin, Fournier, Corre, Corguillé, Bernay and Henry [81] obtained peptides from hydrolysates of white shrimp (*Litopenaeus vannamei*) by-products. The hydrolysis was performed by Protamex enzymes at 50 °C. All three fractions of the resulting peptides had antibacterial activity against *Yersinia ruckeri*, *Bacillus megaterium* and *Edwardsiella tarda*.

Hsu [89] verified the antioxidative properties of peptides obtained from hydrolyzed protein of tuna dark muscle by-product using two commercial enzymes (orientase and protease XXIII) at pH 7.0 and 50 °C and pH 7.5 and 37 °C, respectively, for up to 6 h. The protein hydrolysate was subjected to gel filtration chromatography and the purified fractions of peptides showed antioxidant activity.

Garcia *et al.* [90] extracted peptides showing antioxidant and antihypertensive properties from waste material derived from the processing of cherry, which protein content of seeds was close to 39% of the dried and defatted seed. Peptide extracts obtained by the digestion using two different enzymes recovered highest antioxidant and antihypertensive capacities. In addition, when the authors used 3 and 5 kDa membranes in the ultrafiltration for separation of hydrolysates, the fractions obtained showed high antihypertensive and antioxidant power. Identified peptides in antioxidant fractions by mass spectrum (MS) detection using a quadrupole time-of-flight (Q-TOF) coupled to a High-Performance Liquid Chromatography (HPLC) (HPLC-ESI-Q-TOF) showed less than 10 amino acids and a high number of hydrophobic and aromatic amino acids, which are characteristic features for antioxidant peptides. Some identified peptides in antihypertensive fraction contained proline, which is a characteristic amino acid within antihypertensive peptides.

A recent study using gastrointestinal proteases for digestion of smooth hound by-products investigated the bioactive peptide production. When the enzymes from intestinal extract were used for hydrolysates production, a highest antioxidative activity (IC_50_ value of 1.47 ± 0.07 mg/mL) was obtained using DPPH method. On the other hand, the alkaline protease was able to produced hydrolysate with the highest ACE inhibitory activity (82% ± 1.52% at 2 mg/mL) [91].

Another kind of waste that can be employed to bioactive compound production is the one generated during olive oil manufacturing. The different extraction process used in olive oil production can generate a lot of waste, which can have great environmental impact [85]. In 2013 and 2014, the production of olives in Europe was approximately 6.9 million tons per year, according to the International Olive Council (IOC) [92]. Thus, assuming that 100 kg of fresh olives generate approximately 2 kg of flour with approximately 22% protein, this waste is highly attractive to perform protein hydrolysis and to produce bioactive peptides [85]. The hydrolysis of proteins from olive seed waste by specific enzymes (Alcalase, Thermolysin, Neutrase, Flavourzyme and PTN) at 50 °C, for 2 h, enables it to be transformed into hydrolysates with antioxidant and antihypertensive capacity [78].

Soybean, a legume with high protein content, has become one of the most consumed food in the world; besides being a very cheap source of protein; its consumption has always been associated with health benefits [93]. In addition to oil, a protein-rich soy meal is obtained from soybean processing, which is usually destined for animal nutrition [94]. Peptides with bioactivity against colon, liver and lung cancer cell proliferation were obtained from soybean meal, a by-product of oil extracted from seeds [77].

Several peptides can be obtained from different approaches, including the type of hydrolysis employed and variations of the operational parameters. The type of peptide generated and its property is dependent on the properties of the protein substrate, and particularly the specificity of the enzyme [95,96]. For this reason, bioactive peptides for food and pharmaceutical industrial applications should be obtained either by the proteolytic action of microorganisms on protein-rich waste or by *in vitro* enzymatic hydrolysis of proteins [9].

The enzymatic hydrolysis of protein-rich waste is carried out by proteases, which are obtained from plants, microorganisms and animals for the production of bioactive peptides. Commercial enzymes commonly used for bioconversion of waste into bioactive peptides are Flavourzyme [76], pepsin [83,97], alcalase [97,98], chymotrypsin [98], orientase, protease XXIII [89], protamex [99,100], α-chymotrypsin, neutrase, papain, tripsina [97], among others. Table 2 shows the summary of some bioactive peptides obtained from agro-industrial waste by enzymatic hydrolyses.

The use of commercial enzymes increases the cost of the process. In this sense, non-commercial enzymes, or even microorganisms, have been used for obtaining bioactive biomolecules. In the literature, few studies of the use of microorganisms for the bioconversion of waste have been published: these microorganisms produce proteases capable of degrading recalcitrant proteins such as keratin [80,84,86]. The microorganism *Chryseobacterium* sp. kr6 produces alkaline keratinases for feather protein hydrolysate production with dipeptidyl peptidase-IV, antioxidant and angiotensin-I converting enzyme inhibitory activities [80]. Fakhfakh, Ktari, Haddar, Mnif, Dahmen and Nasri [84] applied *Bacillus pumilus* A1 to the degradation of chicken feathers to yield protein hydrolysates with antioxidant activity. *Bacillus pumilus* A1 was also used for sheep wool-waste biodegradation, in which the hydrolysate had antioxidant activity [86]. The yield obtained by *in vitro* digestion of chicken and wool waste with *Bacillus pumilus* A1 was very high, reaching 98% [84] and 97% [86], respectively.

Regarding non-commercial use of enzymes, extracellular proteases from *Aureobasidium pullulans* were produced, purified and characterized. Purified protease was used for enzymatic hydrolysis of different substrates (marine yeasts, milk and casein). Hydrolyzed *Spirulina* showed high ACE inhibitory and antioxidant activities [101]. Several enzyme preparations commercial and non-commercial were evaluated in hydrolysis of heads and viscera of *Sardinella aurita*. The hydrolysates showed inhibitory activity towards ACE but the alkaline protease extract from the viscera of sardine produced hydrolysate with the highest ACE inhibitory activity [98].

Generally, waste does not undergo pretreatment before enzymatic hydrolysis. In some cases, only the grinding operation unit is applied to the waste [100] and the one with high lipid content, such as chicken breast skins, need a defatting step prior to hydrolysis [102]. The hydrolysis reaction conditions, such as time, temperature, pH and enzyme:substrate ratio, must be optimized to trigger the activity of the enzyme [10]. The hydrolysis reaction ends with heat; afterwards, protein hydrolysates are filtered or centrifuged and bioactive peptides are recovered by purification techniques.

The degree of hydrolysis carried out by endo- and exopeptidases may differ. Efficiency of protein hydrolysis is more pronounced when a combination of endo- and exopeptidases is employed. Usually, the hydrolysis starts with endopeptidase action, and, afterwards, exopeptidase is applied [76,103].

Available waste and by-products are potentially a cheap source of active biomolecules, such as peptides. In addition, no process has been applied on a large scale to obtain bioactive peptides; thus, investments in studies and processes to obtain these biomolecules on a large scale are needed.

## 4. Recovery and Purification Process

Commercially, there has been increasing the interest in producing bioactive peptides due to their therapeutic potential. However, these compounds must be purified for commercial use. Isolation and purification of bioactive peptides are also very important, not only to explore their physicochemical properties, but also to evaluate the bioactivities properties by *in vitro* and *in vivo* assays [10].

The first step to consider in the purification is the purpose of the process. The three factors that influence the development of design industrial processes are the purity, cost effectiveness and process time. It is noteworthy that purity is defined by the final intended use of the product, e.g., 95% purity is required for an *in vitro* diagnosis, whereas 99.998% purity is required for therapeutic application [104]. Therefore, studies of recovery and purification of bioactive peptides should be performed in order to obtain an economically viable product.

Conventional purification of any biotechnological product traditionally involves the following steps [105]:

1. *Removal of insolubles*: Filtration and centrifugation are the principal unit operations used in this segment. Relatively little product concentration or improvement of product quality occurs.
2. *Isolation and concentration of products*: These steps, which are relatively nonspecific, remove materials of widely divergent properties by comparison with the desired product. Appreciable concentration and increase in the product quality usually occur. Adsorption and solvent extraction are typical.
3. *Purification*: This processing technique is highly selective for the product and removes impurities of similar chemical functionality and physical properties. Chromatography, electrophoresis and precipitation are good examples.
4. *Polishing*: The end use of the product dictates the final sequence. Crystallization is often used. Most products must also be dried.

Purification steps may represent a high share of the total production cost of a bioproduct. Therefore, the following rules are useful to ensure the success of the process: keep purification simple, minimize the number of steps and avoid difficult manipulations that will not reproduce; avoid expensive techniques; optimize each step of the process; use reliable techniques and apparatus; bear in mind your objectives, be they high yield, high purity, final scale of operation, and/or reproducibility; and, last but not least, know about the structure, function and properties of the target protein to set up a correct purification strategy [106].

Most protein recuperation and purification processes can be used for separation of bioactive peptides [107]. Before the separation process, ammonium sulfate precipitation, salting out and solvent extraction steps may be performed to remove interferents present in the crude extract, as enzymes, lipids, proteins and other compounds [10].

It is well known that chromatography is the most powerful technique to isolate and purify bioactive peptides. Different chromatographic systems have been developed based on the properties of molecules [108]. Table 3 shows the description of the chromatography methods frequently used for peptide purification.

High-performance liquid chromatography (HPLC) is the technique most used for chromatography methods because of some technical features, such as reproducibility, ease of manipulation and high recovery. In addition, the most important characteristic of this type of chromatographic system is its high resolution achieved even with structurally similar molecules [108].

Reversed-phase chromatography is undoubtedly the most commonly used technique separation for peptides, although ion exchange, size exclusion, affinity and hydrophobic interaction chromatographies have also been applied [4,79,82,87,89,97,110,111,112,113,114,115]. It is noteworthy that, in most studies, a combination of more than one chromatographic technique is used to better separate peptides. For example, in Kim’s study, a novel antioxidant peptide from *Ruditapes philippinarum* was purified by a combination of ultrafiltration, ion exchange chromatography (diethylaminoethyl (DEAE)-Sephacel) and reverse-phase HPLC [114].

Peptides isolated from egg-yolk protein preparation with ACE-inhibitory were purified by a sequence of different methods: ultrafiltration, size exclusion chromatography and reverse-phase HPLC [82]. Lee *et al.* obtained an ACE-inhibitory peptide from enzymatic hydrolysis of chum salmon (*Oncorhynchus keta*) skin [97]. The peptide was purified by size exclusion chromatography (Sephadex G-25) and reversed-phase HPLC (Grom-sil 120 ODS-5 ST column).

Sheih, Fang and Wu [87] obtained a potent antioxidative peptide from hydrolyzed alga protein waste. The peptide was purified by ammonium sulfate precipitation and the precipitate was collected and dissolved in distilled water. Then, the solution was fractionated by size exclusion chromatography (Sephacryl S-100). The fraction with the highest antioxidative activity was subsequently loaded onto a Q-sepharose fast flow column (ion exchange chromatography).

In Xie’s study, a zinc-chelating peptide obtained from hydrolysis of rapeseed meal with alcalase was purified by immobilized-metal affinity chromatography (IMAC-zinc (II)) and gel filtration (Sphadex G-25) [79]. Reversed-phase high-performance liquid chromatography (RP-HPLC) was used to separate the desirable fractions after gel filtration.

Other purification techniques, such as capillary electrophoresis, capillary isoelectric focusing, counter-current chromatography and centrifugal partition chromatography, have also been used for peptide purification [10,116,117,118,119,120,121].

Membrane processes are commonly used processes in large-scale separation of bioactive peptides [14]. These processes are based on the differences in the permeability of the liquid constituents through a membrane. The driving force applied to the mass transport is a partial pressure. The membrane separation processes could be divided into ultrafiltration, microfiltration, reverse osmosis and nanofiltration (Table 4) [122].

Ultrafiltration has routinely been employed to enrich bioactive peptides from protein hydrolysates and to isolate short peptides from high molecular mass residues and enzymes separation [76,80,123,124]. In many studies, ultrafiltration is used for fractioning peptides, and then one or more chromatographic techniques are applied, in sequence, to increase the purity of the fractions [82,100,114,115,125]. In Wu’s study, an enzymatic ultrafiltration reactor was used to hydrolyzed and fractionated peptides with antihypertensive and antimicrobial activities and casein phosphopeptides [125]. A sequence of size exclusion chromatography (SEC), strong cation exchange high-performance liquid chromatography (SCE-HPLC) and reversed-phased high performance liquid chromatography (RP-HPLC) was applied for the peptides purification.

Nanofiltration membranes were also used to separate peptides on the basis of charge interaction with the membranes in addition to size separation, since most peptides contain charged functional groups at a given pH. The combination of membrane processes (UF and NF) is often used in the separation of peptides [100,126,127]. To improve the yield and selectivity of the peptide separation, other driving forces have been used in the membrane process, including electrical potential difference (electrodialysis and electrophoresis) and the combination of electrical potential gradients and pressure (electronanofiltration and electrofiltration) [128,129,130,131,132].

Doyen *et al.* performed the enzymatic hydrolysis of β-lactoglobulin and the simultaneous separation of anionic and cationic peptides generated in an electrodialysis cell with ultrafiltration stacked membranes [128]. Anionic and cationic peptides with hypocholesterolemic, antihypertensive and antibacterial properties were recovered and concentrated.

Nanofiltration and a combination of electrodialysis with ultrafiltration membrane (EDUF) were compared by Langevin *et al.* [132]. Both processes led to different results since nanofiltration was more efficient in terms of mass flux than EDUF comparing same membrane area and the process duration, while EDUF was more efficient for recovery a larger range of peptide molecular weights and to recovery more polar amino acids. The EDUF showed an increase in antioxidant capacities due to better peptide isolation. An increase in antioxidant capacity on H_2_O_2_ degradation assay for the peptides isolated by nanofiltration was also observed. Thus, this work shows that the combination of nanofiltration and EDUF could optimize the separation process and result in more specific peptide fractions.

Ultrafiltration membrane bioreactors have been used for simultaneous enzymatic hydrolysis and isolation of bioactive peptides from a large variety of protein sources. The efficiency of enzyme-catalyzed bioconversion and product yield could be improved using these processes. Besides that, ultrafiltration membrane reactors can easily be scaled up and provide a uniform product containing the desired peptides with specific molecular mass characteristics [14,125,133].

Wang *et al.* compared the efficiency of oligopeptides production by traditional bath enzymatic hydrolysis and an ultrafiltration process coupled with enzymatic hydrolysis [133]. The authors demonstrated that the combination of the ultrafiltration and the enzymatic hydrolysis in the same reactor provides benefits for the oligopeptides process. The ultrafiltration-coupled to enzymatic hydrolysis increased the content of oligopeptides up to 60%, compared to less than 40% using batch enzymatic hydrolysis. Additionally, ultrafiltration-coupled to enzymatic hydrolysis methodology had excellent stability in the molecular weight distribution of the collected peptides at different hydrolysis time and more antioxidant activity than the batch enzymatic hydrolysis.

Most of the purification methods mentioned in this review are effective in laboratories. However, they are no used in large scale for industrial applications due to the process cost effectiveness. Peptide-based products commercialization is still limited due to the high cost of separation and purification techniques and the lack of technologies applicable for industrial scale [14].

A few bioactive peptides have been commercialized in the form of fermented milks. However, industrial-scale production of bioactive peptides is hampered by the lack of suitable technologies. Besides, there is the need to develop technologies that retain or even enhance bioactivity of peptides in food systems [134]. Thus, it is evident that there is need for establishing an efficient, inexpensive and scale-up process to obtain, recover and purify these biomolecules, allowing its application to food and pharmaceutical industries.

Additionally, in order to obtain peptides with bioactive properties and to use them effectively, it is necessary to choose a protocol that involves the choice of the most appropriate co-product, the way it will be obtained and the purification process that will be employed. Furthermore, new technologies, such as nanotechnology, have emerged as a way to enable peptide transport and ensure its functionality during application.

## 5. Nanotechnology

**Figure 2:**
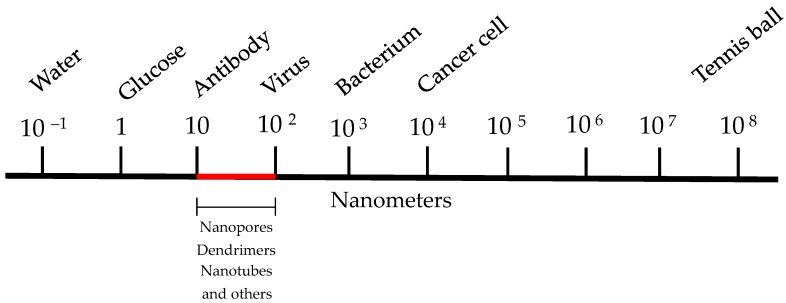
Diagrammatic representation of the size range of material in nanotechnology.

Nanotechnology, in a general way, is the study of the control of matter in the size range of 100 nm or smaller. As a comparison, a hydrogen atom is 0.1 nm in diameter, a lysosome is between 200 and 500 nm, an *Escherichia coli* bacterium is about 2 μm in length and most eukaryotic cells are between 8 and 30 μm in diameter or larger. The magnitude of proteins ranges between 3 and 90 nm; therefore, many enzymes, signaling molecules and receptors are in the nanoscale range [135]. Since most biological processes occur at the nanoscale, nanoparticulate technology has a promising future to develop novel preventive, diagnostic and therapeutic agents, such as bioactive peptides. Figure 2 shows the diagrammatic representation of the size range of materials used in nanotechnology.

The economic impact of nanotech products is in order of trillions of US dollars, and nanobiotechnology has a great share of this market. The sectors that have benefits with the development of products, technics, methodology and processes on nanometric scale are the health, food and agroindustry. The last one represents a fertile field for applications on a large scale. The nanobiotechnology is a reality and the knowledge of biomaterials, biological sciences and engineering, through the union of different research groups on their specific fields, allows the conception of products never considered many years ago. Many nutrients, phytochemicals, enzymes, peptides and other natural compounds can be loaded into biocompatible and biodegradable nanoparticles, which will improve some of their characteristics, such as aqueous solubility, stability, bioavailability, circulation time and target specificity [136]. Functional ingredients, for example bioactive peptides in addition to other important aim biomolecules, are components of an extensive range of industrial products, for instance pharmaceuticals, health-care merchandises, cosmetics, agrochemicals and foods. Functional ingredients are not often applied straight in their pure form. Instead, they are frequently incorporated into some method of delivery system [13].

The interest in studying bioactive peptides using nanotechnology to improve their application has grown impressively in recent years, which shows the importance of cataloging the different uses and interests to advance in this area is ongoing and the information is made available in organized way, as is the case of this review. Table 5 gives some examples of applications involving nanotechnology and peptide in recent years.

The technology of controlled drug delivery is one of the frontiers of science, which involves several multidisciplinary aspects and can greatly contribute to the advance of human health. Delivery systems, often described as drug delivery systems, offer numerous advantages by comparison with other conventional dosages: they provide higher therapeutic efficacy, with progressive and controlled release of the drug from the degradation of the matrix and significantly decrease toxicity and high residence time in the circulation [12]. New strategies include the use of important applications of science colloids, in their various forms, such as multiple and inverse emulsions, microgel, nanogel, liposomes, biodegradable materials, microcapsules, nanocapsules, microparticles, nanoparticles and nanofibers.

It is important to define some terms related to nanoencapsulation, since structures and types diverge greatly. The term nanoparticle is generic and it is used in accordance with the size of the particle that it refers to. Particles smaller than 1 mm are considered nanoparticles, whereas larger particles are called microparticles. The word nanoparticle applied to controlled release is broad and refers to two different forms, nanosphere and nanocapsule structures. Those spheres are called systems in which the drug is homogeneously dispersed or solubilized within the polymeric matrix. Thus, a monolithic system is obtained where a differentiated nucleus cannot be identified. Nanocapsules, on the contrary, are the so-called system reservoirs, where it is possible to identify a distinct core, which may be solid or liquid. In this case, a membrane, generally a polymeric one, encloses the substance and insulates the core from the external environment [135].

A delivery system shows as an essential requirement to be able to perform diverse roles. Primarily, it is used as a vehicle to transport the functional ingredient to the preferred site of action. Then, it may have to shield the functional ingredient from degradation (for example, oxidation) during handling, storage and usage; it preserves the functional ingredient in its active state. Thirdly, the capacity of controlling the release of the functional ingredient has to be controlled, for example, the release rate and the specific environmental conditions that start the release process (pH, ionic strength, temperature, among others). Finally, the delivery system must be well-matched not only with the other constituents in the system, nevertheless similarly with the physicochemical and qualitative characteristics of the final product [147].

Bioactive peptides are viewed as nutraceuticals that can be industrially manufactured and inserted into several foods and drinks. However, peptides could be degraded in the course of digestion, [148] resulting in decreased or probable attenuated bioactivity. Several authors have recently used nanotechnology to improve the use of bioactive peptides. Several examples are described below.

Nanomedicine exploits homing peptides as a way to functionalize free drugs or nanostructured materials applied as drug carriers. Xu *et al.* produced tumor-homing peptides as systematic nanoparticles in order to increase receptor-mediated cell penetrability [149]. The nanoparticulate peptide versions were significantly more effective as mediator of the receptor-dependent uptake than their free equivalents. Their results highlights an additional advantage of nanostructured materials constructed on repetitive building blocks, concerning the multivalent presentation of cell ligands that could facilitate the cell penetration considering drug delivery uses.

Regarding biomedical purposes, several advances have been made by nanotechnology and bioactive peptides. As an example, heparin mimetic peptide amphiphile (HM-PA) nanofibrous network has recently shown to be a promising platform to improve the functionality and transplantation effectiveness of the pancreatic islet *in vitro*, since this procedure is considered a favorable treatment for type 1 diabetes, even though transplantation can reduce the viability and functionality due to loss of integrity and destruction of blood vessel networks. Therefore, it is imperative to afford proper mechanically and biologically helpful environment to improve *in vitro* islet culture as well as the transplantation efficiency [150].

Scaffolds produced with nanotechnology knowledge denote extremely useful methods in peripheral nerve recovery, in addition to in spinal cord recovery. Masaeli *et al.* established peptide functionalized polyhydroxyalkanoate nanofibrous scaffolds to improve Schwann cell activity. Relations between Schwann cells (SCs) and scaffolds were shown as an imperative tool for tissue development in nerve regeneration, since SCs physiologically support the orientation of the growth considering regenerating axons [151]. Therefore, electrospun scaffolds were prepared by combining poly (3-hydroxybutyrate) (PHB) and poly (3-hydroxybutyrate-*co*-3-hydroxyvalerate) (PHBV). They were functionalized with collagen I, or with the following peptides: Gly–Arg–Gly–Asp–Ser (GRGDS), Tyr–Ile–Gly–Ser–Arg–NH_2_ (YIGSR) or Arg–Asn–Ile–Ala–Glu–Ile–Ile–Lys–Asp–Ile (p20), which were neuromimetic peptides able to simulate naturally occurring extracellular matrix (ECM) motifs for nerve regeneration.

Loo *et al.* developed ultrashort peptide nanofibrous hydrogels for the acceleration of the healing of burn wounds [152]. Their ultrashort aliphatic peptides have an innate tendency to self-assemble into helical fibers, forming biomimetic hydrogel scaffolds that are non-immunogenic and non-cytotoxic. These nanofibrous hydrogels accelerated wound closure in a rat model for partial-thickness burns. They also promoted epithelial and dermal regeneration in the absence of exogenous growth factors, achieving 86.2% and 92.9% wound closure respectively, after 14 days. Since the rate of wound closure is inversely correlated with hypertrophic scar formation and infection risks, this peptide hydrogel technology fills a niche neglected by current treatment options. The regenerative properties can be further enhanced by the incorporation of bioactive moieties, such as growth factors and cytokines.

Bagheri *et al.* assessed spray-dried alginate microparticles loud caffeine-loaded and bioactive nanoparticles [153]. Nanoparticles were arranged from antioxidant peptides with the desolvation using ethanol and spread into a sodium alginate solution. The studied showed that caffeine was encapsulated into nanoparticles produced via desolvation of potentially bioactive peptides using ethanol. The peptidic nanoparticles microencapsulated improved the nanoparticles stability toward the imitation gastric digestion slowing down the release of caffeine from the microparticles.

Inductive growth factors as well as regulation of protein-based extracellular matrix components, glycosaminoglycans (GAGs) are responsible for bone tissue regeneration. GAGs establish an important fraction of extracellular matrix and represent a substantial impact on regulating cellular behavior, directly or acting over the encapsulation and presentation of growth factors to the cells. Kocabey *et al.* [154] established nanofibers that stimulate the mineralization by osteogenic cells. Sulfonate and carboxylate groups were used to produce GAGs and collagen mimetic peptide nanofibers. The GAG mimetic peptide nanofibers act together with bone morphogenetic protein-2 (BMP-2), critical growth factor for osteogenic activity. GAG mimicking capability of peptide nanofibers and their interaction with BMP-2 stimulated osteogenic activity and mineralization by osteoblastic cells. Alkaline phosphatase activity, Alizarin red staining and energy dispersive X-ray analysis spectroscopy showed the effectiveness of peptide nanofibers in inducing mineralization. The multifunctional and bioactive microenvironment defined in this work offers osteoblastic cells with osteogenic stimuli comparable to those perceived in native bone tissue.

Balcao *et al.* [155] encapsulated lactoferrin (a whey protein fraction with bioactivity) within a water–oil–water nanoemulsion as potential antimicrobial formulation. Nanoencapsulated lactoferrin and lactoferrin in solution showed inhibitory effect against *Staphylococcus aureus*, *Listeria innocua*, *Bacillus cereus* and *Candida albicans*, but not against Gram negative bacteria, such as *Salmonella* sp., *Escherichia coli* and *Pseudomonas fluorescens*.

Antimicrobial peptide P34 was nanovesicle-encapsulated by Malheiros, Sant′Anna, Utpott and Brandelli [4] and then used in milk as an antilisterial bioactive. The authors aimed to assess the effect of free and nanovesicles-encapsulated BLS P34 against *Listeria monocytogenes* (*L. monocytogenes*) in milk. Thereafter, the preservation of antimicrobial activity was measured over time. The antimicrobial activity of free and encapsulated BLS P34 reduced approximately 50% after four days of storage (4 °C). Afterward, the activity did not show any significant loss up to 21 days. The amount of *L. monocytogenes* in skim and whole milk containing 3200 activity units per mL (AU/mL) of free or encapsulated BLS P34 showed reduced values when compared to the controls without bacteriocin at 30 and 7 °C. Considering a concentration of 1600 AU/mL, free and encapsulated BLS P34 were inhibitory to *L. monocytogenes* in skim milk, comparing with the control at seven days. The conclusion of the work was that nanovesicle-encapsulated and free BLS P34 presented potential use as biopreservative for appliance to milk-derived products.

Meira, Daroit, Helfer, Correa, Segalin, Carro and Brandelli [45] studied bioactive peptides in ovine cheeses from Brazil and Uruguai. Feta-type, Roquefort-type and Pecorino-type cheeses from Brazil, in addition to Pecorino Sardotype and Cerrillano cheeses from Uruguay, were investigated. Antioxidant properties were evaluated using 2,2′-azino-bis-(3-ethylbenzothiazoline)-6-sulfonic acid (ABTS), Thiobarbituric acid reactive substance (TBARS) as antioxidant methods. Scavenging of the cation radical of ABTS oscillated from 32% to 45% for Feta-type cheeses and 87% for Roquefort-type cheese. A comparable trend was detected for the reducing power, that is, WSE from Roquefort-type cheese presented the highest activity among the assessed cheeses. Iron chelating activity was fairly inconstant considering the different WSE; it was higher (50%) for a Pecorino-type cheese. TBARS investigation presented resemblance among most cheese samples, with inhibition values oscillating from 25% to 51%. Scavenging of DPPH radical was detected only for the Roquefort-type cheese. All WSE samples displayed a protuberant inhibition of angiotensin I-converting enzyme, varying from 46% for Feta-type cheese to 80% for Roquefort-type cheese. Results showed that the cheeses studied could be sources of bioactive peptides with diverse ways of action. Nano-ESI-MS/MS method permitted the identification of peptides that may contribute to the bioactivities.

Imran *et al.* proposed the fusion of both concepts to improve bioavailability, that is, antimicrobial peptide (AMP) “nisin” nanoencapsulation and biopolymer immobilizing to produce biodegradable films entrenched with active agent or nano-encapsulated active agent, or both of them. Nanoliposomes were produced using soy-lecitin in a microfluidizer to create an average size of 151 nm, presenting an encapsulation efficiency of 50% [156]. Nisin nano-emulsion (encapsulated and free nisin) films showed effectiveness against *Listeria monocytogenes*. Consequently, it is able to be an operational way to control food pathogen without compromising the physico-chemical characteristics of composite HPMC biodegradable films.

Liposomes containing nisin and BLS P34 were produced in the study of Malheiros, Sant′Anna, Barbosa, Brandelli and Franco [5]. Nisin and BLS encapsulated in P34 PC-1-cholesterol were 218 nm and 158 nm in diameter, zeta potential of −64 and −53 mV, and entrapment efficiency of 88.9% and 100%, respectively. The authors showed that all treatments decreased the population of *L. monocytogenes* compering with the control for 21-day storage of Minas frescal cheese at 7 °C. Nevertheless, nisin and BLS P34 encapsulated in PC-1-cholesterol liposomes were less efficient to control *L. monocytogenes* growth in comparison with free and PC-1 liposome-encapsulated bacteriocins. The best condition considering the inhibitory effect evaluated was observed when the experiment was done using nisin and BLS P34 encapsulated in PC-1 liposomes after 10-day storage.

Andukuri *et al.* developed a biomimetic hybrid nanomatrix combining electrospun polycaprolactone (ePCL) nanofibers with self-assembled peptide amphiphiles (PAs) [157]. The electrospun polycaprolactone nanofibers presented an interconnected nanoporous structure, on the other hand were vulnerable by a lack of surface bioactivity in order to regulate cellular behavior. Transmission electron microscopy proved the uniform coating of self-assembled PA nanofibers on ePCL. Outcomes found by those authors show that this hybrid nanomatrix has pronounced potential use considering cardiovascular implants.

Considering all previously mentioned studies and the development of nanotechnology when used as a tool to increase the application of bioactive peptides, much knowledge has emerged in biotechnological, pharmaceutical, medical, and diagnostic areas as well as others. Points of contact among different areas of knowledge have interspersed within nanobiotechnology. It has also been proven that nanotechnology provides product development that goes beyond the limitations of cost, performance and workmanship, by comparison with conventional production methods.

## 6. Conclusions

Because of several technological and therapeutics properties of bioactive peptides, the production of these biomolecules from protein-rich waste is a promising approach to be exploited by the food and pharmaceutical industry. Currently available waste and by-products are potentially low-price sources for the production of these peptides. Despite the advances achieved by research, further studies are still needed in order to reduce the costs of production, downstream processes and scalability. The use of technologies for entrapment of peptides in nanostructures is an innovative technology for application of these substances more effectively. The advantages of the peptides encapsulated in nanoparticles in relation to free peptide shows that it is necessary to develop new technologies and new materials for entrapment of bioactive peptides in nanostructures, particularly to combat infectious diseases.

## Figures and Tables

**Table 1:** Protein content of agro-industrial waste available to obtain bioactive peptides.

Agro-Industrial Waste	Proteins (%)	Reference
Alga protein	>50.0	[75,83]
By-products of shrimp (*L. vannamei*)	>64.0	[81]
Chicken raw feathers	85.3	[84]
Fish and shellfish	10–23	[8]
Olive stone	>22.0	[78,85]
*Porphyra columbina* residual cake	>27.0	[76]
Sheep raw wool	80.7	[86]
Soybean meal	90–93	[77]

**Table 2:** Peptides from agro-industrial waste by enzymatic hydrolyses with their respective bioactivity and purification techniques.

Agro-Industrial Waste	Bioactivity	Hydrolysis	Techniques Purification	Reference
*Porphyra columbina* residual cake	ACE Inhibitory and antioxidant properties (DPPH)	Acid protease (fungal protease concentrate) + Flavourzyme	Gel filtration chromatography	[76]
*Chlorella vulgaris* waste	Antioxidant and anticancer activities	Pepsin	Ammonium sulfate precipitation, gel filtration, ion exchange chromatography	[83]
Chicken feathers	Antioxidant, ACE- and DPPH-IV inhibitory activities	*Chryseobacterium* sp. kr6	Ultrafiltration, HPLC	[80]
Chicken feathers	Antioxidant activity	*Bacillus pumilus* A1		[84]
Sheep wool	Antioxidant activity	*Bacillus pumilus* A1		[86]
Heads and viscera of sardinelle	ACE-inhibitory activity	Crude enzyme extract from sardine (*Sardina pilchardus*) viscera	Gel filtration	[98]
*Spirulina* (*Arthospira platensis*)	ACE-inhibitory activity	Extracellular proteases from *Aureobasidium pullulans*	Ultrafiltration	[101]
Tuna dark muscle	Antioxidant activity	Orientase and protease XXIII	Gel filtration, two-steps of HPLC	[89]
Residual meat of hard clam	ACE-inhibitory activity	Protamex	Gel filtration	[99]
Chum salmon (*Oncorhynchus keta*) skin	ACE-inhibitory activity	Trypsin	Gel filtration, reversed-phase HPLC	[97]
Atlantic rock crab (*Cancer irroratus*)	Antibacterial activity	Protamex	Micro-, ultra- and nanofiltration, ion exchange chromatography	[100]
Egg-yolk phospholipid extraction	ACE-inhibitory activity	Protease from *Cucurbita ficifolia* fruit pulp	Ultrafiltration, gel filtration and reversed-phase HPLC	[82]

HPLC: high-performance liquid chromatography.

**Table 3:** Principles of the chromatography methods used for bioactive peptide purification.

Method	Principle
Reversed-phase	Based on hydrophobicity. Consists of a stationary phase of lower polarity and a mobile phase of higher polarity.
Ion exchange	The distribution and surface charge of the peptide determines the interaction of charged groups with the surface of the stationary phase.
Size exclusion	Based on separation process according to the size of the peptide relative to pore sizes in the stationary phase. Used primarily in the early stages of purification of the peptide, when performed in multiple steps.
Affinity	Based on the biological specificity of the peptide. Consists of a ligand (small specific biomolecule such as an antibody) that is immobilized in the column. The separation occurs because of highly specific biochemical interactions between the peptide and the ligand.

Reference: Adapted from Espitia *et al.* [109].

**Table 4:** Brief general descriptions of membrane processes.

Method	Description
Ultrafiltration (UF)	UF involves the use of membranes with a molecular weight cutoff in the range of 1–200 kDa and a pore size of approximately 0.01 μm; it is performed at <1000 kPa.
Microfiltration (MF)	MF is a pressure-driven membrane process that involves the use of membranes with pore size of 0.2–2 μm; it can selectively separate particles with molecular weights >200 kDa.
Reverse osmosis (RO)	RO membranes are characterized by a molecular weight cutoff of approximately 100 Da; the process involves pressures 5–10 times higher than those used in UF.
Nanofiltration (NF)	NF separates particles with molecular weights in the range of 300–1000 Da. It allows the rejection of ions based on their diffusion characteristics and charge. NF is capable of removing ions that contribute significantly to the osmotic pressure, thus allowing operation pressures lower than those needed in RO.

Reference: Adapted from Rosenberg [122].

**Table 5:** Application of bioactive peptides using nanotechnology.

Application	Description	Reference
Biodegradable wound dressing nonofiber	Fabrication of nanofibrous P(3HB-*co*-4HB)/collagen peptides construct as potential leave-on wound dressing	[137]
Intracellular delivery	Photosensitizer and polycationic peptide-labeled streptavidin as a nano-carrier for light-controlled protein transduction	[138]
Drug delivery	Ionic graft copolymers to fold and activate ionic peptides through inter-polyelectrolyte nano-assembly	[139]
Implant materials in bone graft substitutes	Peptide decorated nano-hydroxyapatite with enhanced bioactivity and osteogenic differentiation via polydopamine coating	[140]
Brain drug delivery	Brain-targeted delivery of protein using chitosan- and RVG peptide-conjugated, pluronic-based nano-carrier	[141]
Antioxidant Activity	Bioactive peptides/chitosan nanoparticles enhance cellular antioxidant activity of (−)-epigallocatechin-3-gallate	[142]
Cancer management	Nanochemoprevention by encapsulation of (−)-epigallocatechin-3-gallate with bioactive peptides/chitosan nanoparticles for enhancement of its bioavailability	[143]
Drug delivery	Production of porous nano-HA/collagen/PLLA scaffold containing chitosan microspheres for controlled delivery of synthetic peptide derived from BMP-2	[144]
Treatment of atherosclerosis	*In vitro* evaluation of nanocomposite containing bioactive peptides romote endothelialisation by circulating progenitor cells	[145]
Cell therapies	Self-assembly combining two bioactive peptide-amphiphile molecules into nanofibers by electrostatic attraction	[146]
